# Call to action on social cognition measures in clinical research

**DOI:** 10.1016/j.scog.2025.100400

**Published:** 2025-11-18

**Authors:** T.B. Ziermans, M. Hajdúk, A.E. Pinkham, SIRS Social Cognition Research Harmonization Group

**Affiliations:** a Department of Psychology, University of Amsterdam, Amsterdam, the Netherlands; b Department of Psychology, Faculty of Arts, Comenius University in Bratislava, Bratislava, Slovak Republic; c The Centre for Psychiatric Disorders Research, Science Park, Comenius University in Bratislava, Bratislava, Slovak Republic; d Department of Psychiatry, Faculty of Medicine, Comenius University in Bratislava, Bratislava, Slovak Republic; e Department of Psychology, School of Behavioral and Brain Sciences, The University of Texas at Dallas, Richardson, TX, USA

**Keywords:** Social cognition, Assessment, Neuropsychology, Validation, Harmonisation, Crosscultural

## Abstract

**Objective:**

To describe current practices and key barriers in social cognition (SC) assessment, given its central role in psychiatric and neurological disorders and the limitations of existing measures.

**Methods:**

Fifty-two SC experts from 20 countries completed an online survey regarding SC tests and questions about their usage frequency and perceived obstacles.

**Results:**

Only facial emotion recognition tasks were used frequently, while the Hinting task and Reading the Mind in the Eyes Test (RMET) were used by over half of participants. However, 10 experts also urged discontinuation of RMET, mostly due to validity concerns. Major obstacles included lack of culture-appropriate norms and poor psychometric properties.

**Conclusions:**

SC assessment is limited by cultural bias and weak psychometrics. Developing and validating culturally sensitive tools, harmonizing protocols, and securing funding are essential to advance research, enable international trials, and improve clinical outcomes.

## 1 Introduction

Social cognition (SC), broadly referring to the mental capacity to perceive, interpret and respond to social information, is impaired across a broad spectrum of disorders (Cotter et al., 2018; Henry et al., 2016). These deficits are generally associated with poorer real-world functioning and reduced quality of life (Halverson et al., 2019). Therefore, SC measures can serve as an important clinical and research tool to improve our understanding of social difficulties across clinical conditions (Van den Stock et al., 2023).

In recent decades the number and transdiagnostic utilization of SC tests has rapidly expanded to a vast array of clinical and non-clinical specializations, resulting in a rich variety of available instruments and paradigms. Unfortunately, many of these measures suffer from shortcomings such as poor psychometric properties and cultural bias and continue to be used, despite criticism (Bourdage et al., 2024; Pinkham et al., 2018). Consequently, there is an urgent need for harmonized, large-scale, international efforts to develop a universal best practice guideline for SC testing in research and clinical settings.

To address this, we previously conducted an international survey and Delphi consensus study among multidisciplinary SC experts with an international Research Harmonization Group (RHG). The goal was to identify the best transdiagnostic and cross-cultural SC measures to stimulate future harmonization among researchers and clinicians. The outcome showed that relatively few existing measures were considered adequate (Pinkham et al., 2025). Here, we report additional results from the global survey to provide further insights into the status quo of SC research practices and identify key obstacles to improve SC assessment.

## 2 Methods

Online survey content and expert definitions were drafted by authors and further refined with input from RHG-members. The term “expert” for academic researchers was defined as follows:

• research experience (either academic or industry) in the field of psychology, psychiatry, social neuroscience, or an allied discipline for at least 4 years and currently active in one of those fields, AND at least 2 peer-reviewed publications on social cognition, of which at least 1 is as first, second, or senior author, and of which at least 1 has been published in the last 5 years.
• for researchers from non-English speaking countries, articles written in languages other than English qualified if they were published in a peer-reviewed journal.

Non-academic expertise (e.g., clinicians, students, industry team members, or service users) was defined as:

• hands-on experience with, or intricate knowledge of, at least 2 social cognition paradigms.

The online survey was distributed via emailed invitations through the RHG-members' familiarity with experts, as well as supplementary literature searches by graduate students. All RHG members qualified as experts according to the definitions above and were encouraged to complete the survey as well. For detail on additional survey procedures, we refer to Pinkham et al. (2025). The study was approved by the Institutional Review Board (IRB) of The University of Texas at Dallas (IRB-23-177).

The RHG pre-defined a set of 18 well-known SC tests from the literature for which respondents could indicate how often they used them on a 4-point scale. In addition, participants could insert additional tests they use and indicate which three tests they use the most. They were also asked if certain tasks should no longer be used, including their rationale. To evaluate obstacles in SC assessments, participants were asked to rate the extent to which each of 10 predefined limitations posed a problem on a 7-point Likert scale. In addition, they could suggest alternative problems and indicate what they considered to be the biggest obstacles limiting progress in international SC research.

## 3 Results

Fifty-two experts filled out >50 % of items for this final part of the survey. They resided in 20 countries, predominantly in North America (35 %) and Europe (50 %). Most experts identified as male (58 %) and 73 % indicated professor/lecturer (any level) as their profession. Schizophrenia/psychosis was checked by 37 % of experts as their main study population of interest, followed by high-risk for psychosis and general population (both 17 %) and 7 additional subdomains in psychiatry or neurology. For additional detail on respondent characteristics, see Supplementary Table 1.

### 3.1 Frequency of use

Table 1 gives an overview of the user frequency of common SC measures. Only facial emotion recognition tasks (such as ER-40) are used often by experts, and more than half of participants reported using the Hinting task and Reading the Mind in the Eyes Test (RMET) at least occasionally. For the 15 other included tasks, most experts indicated that they never use them. Finally, 16 experts indicated some SC tasks should no longer be used of whom10 mentioned the RMET. This was motivated by issues with construct validity and confounders (e.g., the influence of vocabulary).

**Table 1:** Central tendency measures for use of common social cognition measures.

	N	Mean	Med	SD	#Top3
Emotion Recognition - 40 (ER40), or equivalent ER task	52	2.52	3	1.00	25
Hinting Task	52	2.17	2	1.04	8
Reading the Mind in the Eyes Test (RMET/Eyes Task)	51	2.16	2	0.88	10
The Awareness of Social Inferences Test (TASIT)	52	1.79	1	0.96	8
Mayer-Salovey-Caruso Emotional Intelligence Test (MSCEIT)	52	1.77	1	0.98	8
Faux Pas Task	51	1.63	1	0.75	5
Cartoon Theory of Mind (CToM)	52	1.62	1	0.89	7
Ambiguous Intentions and Hostility Questionnaire (AIHQ)	52	1.54	1	0.92	4
Bell Lysaker Emotion Recognition Test (BLERT)	50	1.46	1	0.71	3
Empathic Accuracy Task (EAT)	50	1.42	1	0.79	5
Trustworthiness Task	52	1.37	1	0.77	1
Mini Profile of Nonverbal Sensitivity (MiniPONS)	52	1.35	1	0.79	1
Emotion In Biological Motion Task (EBM)	52	1.25	1	0.65	3
Point-light walkers	52	1.19	1	0.56	1
Relationships Across Domains Task (RAD)	52	1.17	1	0.51	1
Intentionality Bias Task (IBT)	50	1.16	1	0.42	0
Social Attribution Task, Multiple Choice (SAT-MC)	51	1.16	1	0.46	1
Observable Social Cognition Rating Scale (OSCARS)	52	1.15	1	0.46	1

Note. This table shows frequency scores (mean, median) and their standard deviation (SD) for use of common social cognition tests by experts. Scores were based on a 4-point scale (1 = Never, 2 = Rarely, 3 = Often, 4 = Always). The final column shows how often a test was included in the top 3 of SC tests participants used most frequently.

### 3.2 Obstacles

Table 2 shows how experts evaluated the 10 pre-defined problems within SC assessment. While all obstacles were considered moderately problematic on average, the highest mean scores were reserved for two related issues concerning cross-cultural applicability, i.e., the availability of appropriate norms and the availability of paradigms that can be used internationally. Regarding the biggest obstacle for progression in international SC research, the diverse answers were categorized based on author consensus (see Supplemental Table 2). Most comments and themes aligned with obstacles mentioned in Table 2, with insufficient test norms and validation, as well as cultural/language adaptations standing out. However, two novel themes that were reported were lack of funding and collaboration opportunities as well as theoretical/conceptual ambiguity.

**Table 2:** Central tendency measures for perceived obstacles in using social cognition measures.

	N	Mean	Med	SD
Lack of normative data and/or culture-specific norms	51	4.94	5	1.50
Problems for cross-cultural comparisons (e.g., stimuli/language)	50	4.74	5	1.60
Poor ecological validity	51	4.65	5	1.41
Weak psychometric properties	51	4.49	5	1.65
Floor/ceiling effects/limited sensitivity to capture differences	51	4.35	5	1.41
Low clinical utility (e.g., for differential diagnosis, treatment)	51	4.12	4	1.74
Utility for repeated administrations (e.g., in RCTs)	51	4.08	4	1.50
Lack of support for associations with functional outcomes	51	3.90	4	1.76
Too lengthy or cumbersome	51	3.78	4	1.40
Limited availability of test in my country	50	3.30	2.5	2.22

Note. This table shows frequency scores (Mean, Median) and their standard deviation (SD) for perceived obstacles in using social cognition tests by experts. Scores were based on a 7-point Likert-scale (1 = Not at all; 7 = Very) and indicated to what extent an obstacle was considered problematic.

## 4 Discussion

The findings from our global survey show that SC assessment is currently narrow and likely constrained by several perceived barriers. Straight-forward emotion perception tasks such as the ER-40 are the only type of measurement that is commonly used among experts, followed by tests measuring mentalization and empathy. Notably, the RMET is both one of the most widely used and most frequently criticized social cognition measures. In our survey, 19 % of experts recommended discontinuing its use, citing challenges in interpretation and confounding influences, such as verbal ability. These findings echo methodological criticisms from the literature (Higgins et al., 2025). Yet they do not appear to deter even narrowly defined SC experts from using RMET or other poorly evaluated SC tasks. This ambivalent attitude in the field highlights a lack of consensus, perhaps driven by insufficient theoretical/conceptual clarity on what SC tests measure (Eikelboom et al., 2025; Quesque et al., 2024).

The most prominently endorsed barriers were the lack of culture-appropriate norms and paradigms as well as poor psychometric properties. There is a strong need for funding to develop psychometrically sound and ecologically valid SC tools. Cultural considerations for such tests can be advanced by systematically developing culture-neutral, culture-sensitive, and culture-adapted tests, each accompanied by robust normative data both within and across diverse ethnocultural groups to ensure validity, fairness, and comparability. Meanwhile, it is important to remain mindful that social stimuli are interpreted within diverse sociocultural contexts and to consider modular assessment batteries to better balance cross-cultural and culture-specific elements. Together, these are essential foundations for large-scale (international) clinical trials using harmonized protocols. Even in well-powered trials with effective interventions, inadequate measurement tools may obscure treatment effects and hinder detection of meaningful change, e.g., in functioning or quality of life (Eddy, 2019). We therefore recommend prioritizing the validation of core measures and systematically adapting them across cultures before expanding to new tools.

Dedicated funding and sustained international collaboration are required to improve norm and test development. Commercial publishers and online platforms may contribute, but academic leadership, focused on both international and non-academic partnerships, is essential. We also advocate for targeted funding schemes to support international consortia.

While modern technologies enable large-scale measurement initiatives, these efforts cannot succeed without proper financial support. Clinicians require precise and reliable norms and valid tools for accurate decision-making and monitoring. Basic research should guide assessment choices, while clinical feedback should inform further refinement. Strengthening collaboration between academia and clinicians is key to bridging the research-practice gap.

In conclusion, our survey of global SC experts emphasizes the need for, and further amplifies recent calls for intensified international collaborative efforts to develop more applicable and psychometrically sound gold standard measures for SC (Corbera et al., 2025). Without these steps, it will be difficult to test the efficacy and effectiveness of SC interventions in large scale international clinical trials or routinely use SC assessment in clinical practice.

## Consortia

The SIRS Social Cognition Research Harmonization Group:

Co-authors:

Maxime Bertoux^6^, Kelsey A. Bonfils^7^, Anne M. Buunk^8^, Clare M. Eddy^9^, Anne-Kathrin Fett^10,11^, Michal Hajdúk^2,3,4^, Monica Mazza^12^, Urvakhsh Meherwan Mehta^13^, Lindsay D. Oliver^14^, David L. Penn^15^, Amy Pinkham^5^, Tamsyn Van Rheenen^16,17^ and Tim Ziermans^1^

^6^ Lille Neuroscience and Cognition, Inserm, University Lille, CHU Lille, Lille, France.

^7^ Department of Psychology and Neuroscience, The University of North Carolina at Chapel Hill, Chapel Hill, NC, USA.

^8^ Department of Neurology, Unit Neuropsychology, University Medical Center Groningen, University of Groningen, Groningen, The Netherlands.

^9^ Research and Development, Birmingham and Solihull Mental Health NHS Foundation Trust, and College of Medicine and Health, University of Birmingham, Birmingham, UK.

^10^ Department of Psychology, City St George's, University of London, School of Health & Medical Sciences, London, UK.

^11^ Department of Psychosis Studies, Institute of Psychiatry, Psychology and Neuroscience, King's College London, London, UK.

^12^ Department of Biotechnological and Applied Clinical Sciences, University of L'Aquila, L'Aquila, Italy.

^13^ Department of Psychiatry, National Institute of Mental Health and Neuro-Sciences (NIMHANS), Bangalore, India.

^14^ Campbell Family Mental Health Research Institute, Centre for Addiction and Mental Health, Toronto, ON, Canada; Department of Psychiatry, University of Toronto, Toronto, ON, Canada.

^15^ Department of Psychiatry, The University of North Carolina at Chapel Hill, Chapel Hill, NC, USA.

^16^ Department of Psychiatry, University of Melbourne, Faculty of Medicine, Dentistry and Health Sciences, Melbourne, VIC, Australia.

^17^ Centre for Mental Health and Brain Sciences, Swinburne University of Technology, Sydney, Melbourne, VIC, Australia.

Non-author collaborators:

Minji Bang^18^, Bodi Bodenhamer^19^, Raymond C. K. Chan^20,21^, Beshaun Davis^22^, Ana Flores^23^, Taeyoung Lee^24^, Lucy Livingston^25,26^, Skye McDonald^27^, Arundati Nagendra^28^, Bram-Sieben Rosema^29^, Julia Sheffield^30^, Andrew Spink^31^, Tamara Tavares^32^

^18^ Department of Psychiatry, CHA Bundang Medical Center, CHA University School of Medicine, Seongnam, Republic of Korea.

^19^ Department of Psychiatry, School of Medicine, The University of North Carolina at Chapel Hill, Chapel Hill, NC, USA.

^20^ Neuropsychology and Applied Cognitive Neuroscience Laboratory, CAS Key Laboratory of Mental Health, Institute of Psychology, Chinese Academy of Sciences, Beijing, China.

^21^ Department of Psychology, The University of Chinese Academy of Sciences, Beijing, China.

^22^ Department of Psychiatry, University of Maryland School of Medicine, Baltimore, MD, USA.

^23^ Silver School of Social Work, New York University, New York, NY, USA.

^24^ Department of Psychiatry, Kyungpook National University School of Medicine, Daegu, Republic of Korea.

^25^ Department of Psychology, Institute of Psychiatry, Psychology and Neuroscience, King's College London, Birmingham, UK.

^26^ Neuroscience and Mental Health Innovation Institute, Cardiff University, Birmingham, UK.

^27^ School of Psychology, University of New South Wales, Sydney, NSW, Australia.

^28^ Schizophrenia &Psychosis Action Alliance, Alexandria, VA, USA.

^29^ HAMLETT study and “Kenniswerkplaats Onbegrepen Gedrag”, NHL Stenden Leeuwarden, and Expert by Experience for Anoiksis, Patiënt Association for Susceptibility for Psychosis, Leeuwarden, The Netherlands.

^30^ Department of Psychiatry and Behavioral Sciences, Vanderbilt University Medical Center, Nashville, TN, USA.

^31^ Noldus Information Technology BV, Wageningen, The Netherlands.

^32^ Department of Psychology, The Hospital for Sick Children, York University, Toronto, ON, Canada.

## CRediT authorship contribution statement

**T.B. Ziermans:** Conceptualization, Data curation, Formal analysis, Funding acquisition, Investigation, Methodology, Visualization, Writing – original draft. **M. Hajdúk:** Conceptualization, Data curation, Formal analysis, Funding acquisition, Investigation, Methodology, Writing – review & editing. **A.E. Pinkham:** Conceptualization, Data curation, Formal analysis, Funding acquisition, Investigation, Methodology, Project administration, Writing – review & editing.

## Declaration of competing interest

The authors declare that they have no known competing financial interests or personal relationships that could have appeared to influence the work reported in this paper.

## Appendix A Supplementary data

Supplementary tables Image 1

Supplementary material Image 2

## References

[bb0005] Bourdage R., Narme P., Neeskens R., Papma J., Franzen S. (2024). An evaluation of cross-cultural adaptations of social cognition testing: a systematic review. Neuropsychol. Rev..

[bb0010] Corbera S., Kurtz M.M., Achim A.M. (2025). International perspective on social cognition in schizophrenia: current stage and the next steps. Eur. Psychiatry.

[bb0015] Cotter J., Granger K., Backx R., Hobbs M., Looi C.Y., Barnett J.H. (2018). Social cognitive dysfunction as a clinical marker: a systematic review of meta-analyses across 30 clinical conditions. Neurosci. Biobehav. Rev..

[bb0020] Eddy C.M. (2019). What do you have in mind? Measures to assess mental state reasoning in neuropsychiatric populations. Front. Psychiatry..

[bb0025] Eikelboom W.S., van den Berg E., Beauchamp M.H. (2025). Providing a taxonomy for social cognition: how to bridge the gap between expert opinion, empirical data, and theoretical models. J. Psychiatry Neurosci..

[bb0030] Halverson T.F., Orleans-Pobee M., Merritt C., Sheeran P., Fett A.K., Penn D.L. (2019). Pathways to functional outcomes in schizophrenia spectrum disorders: meta-analysis of social cognitive and neurocognitive predictors. Neurosci. Biobehav. Rev..

[bb0035] Henry J.D., von Hippel W., Molenberghs P., Lee T., Sachdev P.S. (2016). Clinical assessment of social cognitive function in neurological disorders. Nat. Rev. Neurol..

[bb0040] Higgins W.C., Kaplan D.M., Deschrijver E., Ross R.M. (2025). Why most research based on the Reading the Mind in the Eyes Test is unsubstantiated and uninterpretable: a response to Murphy and Hall (2024). Clin. Psychol. Rev..

[bb0045] Pinkham A.E., Harvey P.D., Penn D.L. (2018). Social cognition psychometric evaluation: results of the final validation study. Schizophr. Bull..

[bb0050] Pinkham A.E., Hajdúk M., Ziermans T., SIRS Social Cognition Research Harmonization Group (2025). Harmonizing cross-cultural and transdiagnostic assessment of social cognition by expert panel consensus. Schizophrenia.

[bb0055] Quesque F., Apperly I., Baillargeon R. (2024). Defining key concepts for mental state attribution. Commun. Psychol..

[bb0060] Van den Stock J., Bertoux M., Diehl-Schmid J. (2023). Current potential for clinical optimization of social cognition assessment for frontotemporal dementia and primary psychiatric disorders. Neuropsychol. Rev..

