# Supplementary material for: Call to action on social cognition measures in clinical research
Source: Schizophr Res Cogn. 2025 Nov 18;43:100400. doi: 10.1016/j.scog.2025.100400 (PMC12661152; doi:10.1016/j.scog.2025.100400)
Supplement: Supplementary file 1 — Supplementary tables [file mmc1.docx]

**Supplemental Materials**

Table 1.

*Characteristics of expert survey respondents*

|  | N | % |
| --- | --- | --- |
| Country of Residence |  |  |
| Australia | 1 | 2 |
| Brazil | 1 | 2 |
| Canada | 1 | 2 |
| Chile | 1 | 2 |
| China | 1 | 2 |
| Denmark | 2 | 4 |
| France | 1 | 2 |
| Germany | 3 | 6 |
| Greece | 1 | 2 |
| Hong Kong | 2 | 4 |
| Italy | 1 | 2 |
| Japan | 1 | 2 |
| Netherlands | 10 | 19 |
| Norway | 1 | 2 |
| Poland | 1 | 2 |
| Slovak Republic | 3 | 6 |
| South Korea | 1 | 2 |
| Spain | 2 | 4 |
| United Kingdom | 2 | 4 |
| United States | 17 | 33 |
| Gender Identity |  |  |
| Man | 30 | 58 |
| Woman | 20 | 39 |
| Not indicated | 2 | 4 |
| Age |  |  |
| 21-24 | 1 | 2 |
| 25-29 | 2 | 4 |
| 30-34 | 7 | 14 |
| 35-39 | 5 | 10 |
| 40-44 | 10 | 19 |
| 45-49 | 10 | 19 |
| 50-54 | 4 | 8 |
| 55-59 | 4 | 8 |
| 60-64 | 3 | 6 |
| 65-69 | 4 | 8 |
| 70+ | 1 | 2 |
| Prefer not to answer | 1 | 2 |
| Organizational Affiliation(s)* |  |  |
| University | 35 | 67 |
| University Medical Center | 23 | 44 |
| Research Institute (non-academic) | 1 | 2 |
| Commercial Industry | 1 | 2 |
| Other | 2 | 4 |
| Position(s)* |  |  |
| Professor/Lecturer (any level) | 38 | 73 |
| Researcher | 20 | 39 |
| Clinician | 10 | 20 |
| Other | 2 | 4 |
| Population(s) of Study* |  |  |
| Schizophrenia/psychosis | 19 | 37 |
| High-risk for psychosis | 9 | 17 |
| Depression | 3 | 6 |
| Bipolar disorder | 6 | 12 |
| Autism and neurodevelopmental disorders | 2 | 4 |
| Neurodegenerative diseases | 4 | 8 |
| Traumatic brain injuries | 4 | 8 |
| Cerebrovascular accident | 5 | 10 |
| General population | 9 | 17 |
| Other | 1 | 2 |

* Respondents could select multiple options, and thus percentages may add up to > 100.

Table 2.

*Expert opinions on biggest obstacle limiting progression in internation social cognition research*

| **Category** | **Definition** | **Number of Responses** | **Representative Quote** |
| --- | --- | --- | --- |
| Norms & Validation | Challenges due to lack of normative data, poor psychometric properties, limited ecological validity, and insufficient clinical applicability. | 13 | “We don’t really know the psychometric properties of many of the measures in many of the relevant countries.” |
| Cultural/Language Adaptation | Difficulties in adapting tasks across languages and cultural contexts while maintaining comparability. | 12 | “I think it’s the cultural adaptation of social cognition tests... far more problematic when tests tap into high level cultural understanding.” |
| Test Content & Design | Issues in the development and adaptation of social cognition measures, such as outdated tasks, poor sensitivity, and design limitations. | 7 | “Ceiling effects in TASIT. Lack of instruments with sensitivity to change.” |
| Funding & Collaboration | Barriers related to insufficient funding, lack of international projects, and limited collaboration across sites. | 6 | “Lack of collaboration across sites. Especially US and rest of the world.” |
| Theoretical/Conceptual Ambiguity | Lack of consensus on definitions, theoretical models, and conceptual clarity in the field. | 4 | “Lack of consensus regarding terminology…Lack of empirical models.” |
| Implementation Challenges | Barriers related to technology adoption, siloed research practices, and overemphasis on Western tasks. | 3 | “Developments of new tools/technology goes so fast, that it is hard to keep up with proper validation & implementation.” |
