# Supplementary material for: Call to action on social cognition measures in clinical research
Source: Schizophr Res Cogn. 2025 Nov 18;43:100400. doi: 10.1016/j.scog.2025.100400 (PMC12661152; doi:10.1016/j.scog.2025.100400)
Supplement: Supplementary file 2 — Supplementary material [file mmc2.pdf]

Part 3: Tasks that are used in current research projects

Progress:

Please indicate how often you use the following social cognitive tasks:

	Never	Rarely	Often	Always
Hinting Task	◯	◯	◯	◯
Reading the Mind in the Eyes Task (RMET/Eyes Task)	◯	◯	◯	◯
The Awareness of Social Inferences Test (TASIT)	◯	◯	◯	◯
Emotion Recognition - 40 (ER40), or equivalent simple emotion recognition task	◯	◯	◯	◯
Bell Lysaker Emotion Recognition Test (BLERT)	◯	◯	◯	◯
Trustworthiness Task	◯	◯	◯	◯
Ambiguous Intentions and Hostility Questionnaire (AIHQ)	◯	◯	◯	◯
Intentionality Bias Task (IBT)	◯	◯	◯	◯
Social Attribution Task, Multiple Choice (SAT-MC)	◯	◯	◯	◯
Mini Profile of Nonverbal Sensitivity (MiniPONS)	◯	◯	◯	◯
Relationships Across Domains Task (RAD)	◯	◯	◯	◯
Observable Social Cognition Rating Scale (OSCARS)	◯	◯	◯	◯
Mayer-Salovey-Caruso Emotional Intelligence Test (MSCEIT)	◯	◯	◯	◯
Emotion In Biological Motion Task (EBM)	◯	◯	◯	◯
Point-light walkers	◯	◯	◯	◯
Cartoon Theory of Mind (CToM)	◯	◯	◯	◯
Faux Pas Task	◯	◯	◯	◯
Empathic Accuracy Task	◯	◯	◯	◯
Other task (please describe below)	◯	◯	◯	◯

Please describe the additional social cognitive task(s) you use: ______________________

Please indicate the three social cognition measures that you use most in your research and/or clinical work:

1. ______________________

2. ______________________________

3. ______________________________

Do you believe there are any tasks that should NO LONGER be used to assess social cognition?

○ Yes
○ No

If yes, please state the task and a brief rationale for your opinion.

____

Have you been involved in any cross-cultural comparisons of social cognition?

○ Yes
○ No

If yes, please describe briefly.

____

If you are based outside of US/UK, are measures recommended by the Social Cognition Psychometric Evaluation project (SCOPE) - BLERT, ER-40 or equivalent, Hinting task - available in your country?

○ Yes
○ No
○ Other (please elaborate below)

Please elaborate on the availability of SCOPE measures in your country.

____

To what extent do the following limitations present a problem in our current social cognition measures according to you?

	1: Not at all	2	3	4	5	6	7: Very
Weak psychometric properties (low reliability, factor structure of tasks etc.)	○	○	○	○	○	○	○
Lack of support for associations with functional outcomes	○	○	○	○	○	○	○
Low clinical utility (e.g., for differential diagnosis, treatment planning, etc.)	○	○	○	○	○	○	○
Floor/ceiling effects and/or limited sensitivity to capture differences	○	○	○	○	○	○	○
Utility for repeated administrations in RCTs including lack of alternate forms	○	○	○	○	○	○	○
Poor ecological validity	○	○	○	○	○	○	○
Too lengthy or cumbersome	○	○	○	○	○	○	○

Problems regarding cross-cultural comparisons (e.g., stimuli are not appropriate, language differences, etc.)	○	○	○	○	○	○	○
Limited availability of test in my country	○	○	○	○	○	○	○
Lack of normative data and/or culture-specific norms	○	○	○	○	○	○	○
Other (please specify below)	○	○	○	○	○	○	○

Please describe other limitation(s): ______________________

In your view, what are the biggest obstacles limiting progress in international social cognition research? ______________________

If you are a clinician, in your view, what are the biggest obstacles limiting implementation of social cognition assessment into clinical practice? ______________________

If you have any other suggestions, please feel free to include them here. ______________________
